# A Novel Thoracoabdominal Aorta CTA-based Nomogram Model to Identify Ideal Candidates for Transradial Approach Chemoembolization in Patients with Liver Cancer

**DOI:** 10.7150/jca.73678

**Published:** 2022-07-04

**Authors:** Miao Li, Feng Zhang, Shen-Xin Lu, Yan Shan, Peng-Ju Xu, Ying-Ting Zhou, Ying-E Zhu, Zheng-Gang Ren, Bi-Wei Yang, Xin Yin

**Affiliations:** 1 Liver Cancer Institute, Zhongshan Hospital, Fudan University, 136 Yi Xue Yuan Road, Shanghai 200032, China.; 2 National Clinical Research Center for Interventional Medicine, 136 Yi Xue Yuan Road, Shanghai 200032, China.; 3 Department of Radiology, Zhongshan Hospital, Fudan University, 136 Yi Xue Yuan Road, Shanghai 200032, China.

**Keywords:** thoracoabdominal aortic CTA, transradial approach chemoembolization, nomogram, liver cancer

## Abstract

**Background:** High technical complexity limits the wide use of transradial approach (TRA) chemoembolization in the management of liver cancer. We sought to construct a thoracoabdominal aorta CTA-based nomogram model to identify ideal candidates for TRA chemoembolization in patients with liver cancer.

**Methods:** Patients who had received thoracoabdominal aorta CTA before TRA chemoembolization from 2018 to 2020 were retrospectively enrolled and randomly divided into a training set and a validation set. The clinical characteristics and CTA features were collected to build a clinical model. Univariate and multivariate analyses were used to identify significant clinical-radiological variables. A CTA-based nomogram model was constructed by using multivariate logistic regression analysis. The predictive performance, as well as discrimination efficacy of the model, was evaluated by ROC analysis and calibration plot.

**Results:** Vascular variation (*P*=0.028), Myla classification (*P*=0.030), length from left subclavian artery to the left subclavian artery (*P*=0.017), and angle between common hepatic artery and abdominal aorta (*P*=0.017) were identified as important factors associated with the technical complexity of TRA chemoembolization, indicated by fluoroscopy time of the total procedure. The CTA-based nomogram model was established by these abovementioned variables, which demonstrated good predictive ability in both the training cohort (AUC=0.929) and validation cohort (AUC= 0.769), with a high C-index of 0.928 and 0.827 respectively. Moreover, satisfactory calibrations were confirmed by the Hosmer-Lemeshow test with *P* values of 0.618 and 0.299 in the training cohort and validation cohort.

**Conclusion:** Our study constructs a novel CTA-based nomogram, which can serve as a useful tool to identify ideal candidates for TRA chemoembolization in patients with liver cancer.

## 1. Introduction

Chemoembolization treatments, including transcatheter arterial embolization (TAE) and transcatheter arterial chemoembolization (TACE), are proven effective in primary and secondary liver cancer 1, 2. Currently, transfemoral approach (TFA) is the most commonly used technique in the treatment of liver cancer 2, 3. In TFA chemoembolization, patients are required to maintain a supine position for 12-24 hours to avoid femoral bleeding, which increases the risks of thrombotic diseases and affects the comfort and quality of life 4.

Since the transradial approach (TRA) to coronary angiography and intervention firstly emerged more than two decades ago, it has become a preferred alternative approach to traditional TFA in cardiovascular centers 5. In coronary angiography and intervention, TRA has shown great advantages over TFA with regards to lower bleeding rate, fewer complications of access sites, and lower risk of adverse events or death 6-11. In recent years, TRA chemoembolization has been introduced as a new approach in the management of liver cancer, especially in experienced interventional centers. Relevant studies 2-5, 12 have shown that the use of TRA chemoembolization is safe, well-tolerated, and associated with rare complications. Moreover, patients treated with TRA usually experience less periprocedural pain and shorter recovery time without significant differences in radiation exposure or procedure length, in comparison with those treated with TFA 12, 13. However, high technical complexity and slow learning curve limit the wide use of TRA in most interventional centers 14. Differences in the morphology of the thoracic and peritoneal vessels also make it difficult for interventionalists to implement TRA chemoembolization for some patients, so TRA chemoembolization is not always suitable for all the patients. It is necessary to develop a clinical tool to predict the technical complexity of TRA chemoembolization in individual patients, allowing for objective selection of good candidates for TRA chemoembolization and guiding clinical decision-making.

With the development of imaging technology, three-dimensional vascular reconstructions of thoracoabdominal aortic angiography computed tomography (CTA) scan allow interventionalists to investigate the characteristics of patients' thoracic and celiac blood vessels before treatment. Herein, we established a thoracoabdominal aorta CTA-based nomogram model, hoping to evaluate the technical complexity of TRA chemoembolization before TACE treatment and guide clinical decision-making.

## 2. Materials and Methods

### 2.1 Study population

From Jan 2018 to Dec 2020, patients with liver cancer who received TRA chemoembolization were screened. Patients were included if they met the following criteria: (1) over 18 years old; (2) diagnosed with primary or secondary liver cancer; (3) performance status (PS) classified as 0-1; (4) underwent at least one planned TRA chemoembolization; (5) received thoracoabdominal aortic CTA examination before TRA chemoembolization; (6) liver function (Child-Pugh) classified as A or B; (7) with adequate renal function: serum creatinine ≤2.0 mg/dL; (8) agreed and signed informed consent. Patients were excluded if they met the following criteria: (1) had severe cardiopulmonary dysfunction; (2) be allergic to contrast agents or didn't undergo thoracoabdominal aortic CTA examination; (3) with incomplete laboratory tests or imaging records. Patients were 1:1 assigned to the training cohort and validation cohort by the random numbers generated by the software Stata.

This study was approved by the institutional review board (B2020-153R) and complied with the standards of the Declaration of Helsinki and current ethical guidelines.

### 2.2 Clinical and radiological variables

**Figure 1:**
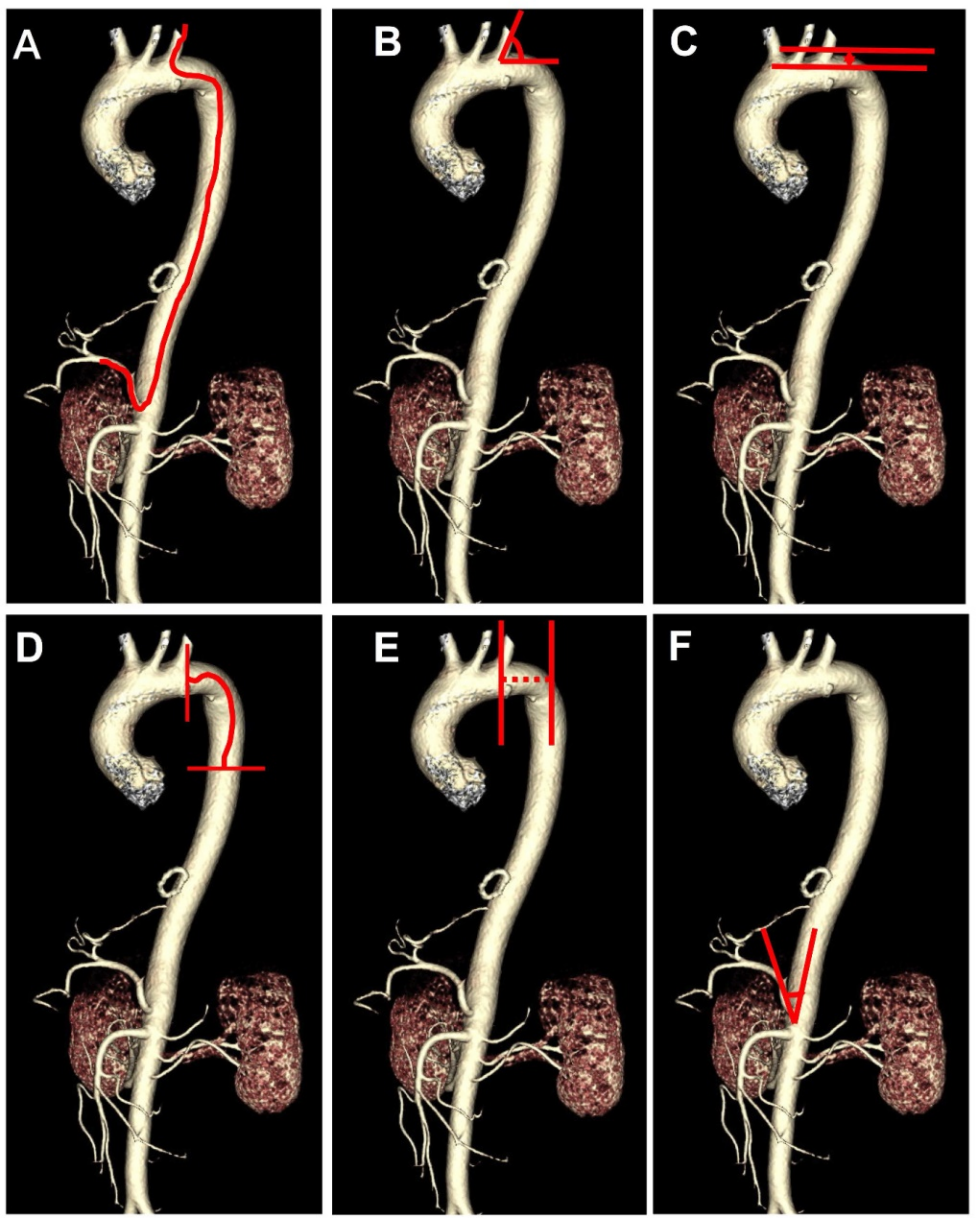
Schematic diagram of the route from the left subclavian artery to the hepatic artery (A) and the angiography computed tomography (CTA) features including the angle between left subclavian artery and aortic arch (B), Myla classification (C), the length from left subclavian artery to thoracic aorta (D), distance from left subclavian artery to thoracic aorta (E), the angle between common hepatic artery and abdominal aorta (F).

The following clinical data were collected, including age, sex, height, weight, medical comorbidities, tumor types, alpha fetal protein (AFP), angiography catheter type, CTA presentations, and total fluoroscopy time. The CTA presentations include vascular variation, Myla classification 15, distance from the left subclavian artery (LSA) to the thoracic aorta (TA), length from LSA to TA, the angle between LSA and aortic arch, the angle between the common hepatic artery (CHA) and abdominal aorta. Vascular variation was divided into 3 groups: none, mild, and severe. Mild vascular variation was defined as: (1) abnormal hepatic/tumor supply vessels from the superior mesenteric artery, left hepatic artery, or phrenic artery, without vascular distortion or deformity; (2) a slightly distorted hepatic artery or slender tumor supply vessel which is unnecessary to change operative catheters. Severe vascular variation was defined as: (1) abnormal hepatic/tumor supply vessels combined with vascular distortion or deformity; (2) distorted hepatic artery or slender tumor supply vessels requiring for changing operative catheters. The schematic diagram of the CTA performances was shown in Figure 1. The imaging parameters abovementioned were measured by two imaging specialists with more than 10 years of working experience.

### 2.3 Procedure

TRA chemoembolization was performed by a group of interventionalists with more than 10 years of working experience. During TRA procedures, patients were supine on the angiography table with their wrists hyperextended. Under local anesthesia with 1% lidocaine, a 5-Fr vascular introducer sheath was inserted into the radial artery. An appropriate amount of vasodilator cocktail (2.8mL) consisting of 1000 IU heparin, 20 mg lidocaine, and 0.2 mg nitroglycerin was injected through the vascular sheath to prevent thrombosis and vasospasm. Angiographic catheters and ultra-selective microcatheters were selected according to patients' vascular conditions. Chemotherapeutic agents and embolic materials were used according to the institutional protocol described elsewhere 16. The dosage of chemoembolization drug was determined by tumor burden, vascularity, and patients' liver function reserve. Generally, Oxaliplatin (100-150 mg) and/or 5- fluorouracil (500-1000 mg) were infused, followed by an injection of a mixture of epirubicin (10-60 mg) or pirarubicin (10-50 mg) and lipiodol (5-20 mL). In some cases, if lipiodol embolization was insufficient, gelatin sponge particles would be used for further embolization. At the end of the procedure, a compression device was placed over the radial access site for hemostasis.

### 2.4 Catheter selection

Commonly, a 4-Fr, 125 cm catheter, and a standard 0.035-inch × 180 cm hydrophilic wire were used for TRA chemoembolization. Based on aortic or hepatic artery morphology, angiographic catheters such as MPA (Johnson & Johnson, USA), Cobra (TERUMO, Japan), and TIG (TERUMO, Japan) catheter could be selected.

### 2.5 Statistics

Continuous variables were presented as median and interquartile range (IQR), whereas categorical variables were expressed as counts with percentages. Univariate and multivariate ordinal logistic regression analyses were applied to identify independent risk factors predicting total fluoroscopy time in the training cohort. The total fluoroscopy time was presented as an ordinal categorical variable based on the cut-off values of 3 and 6 minutes. All the variables in the univariate analysis with *P*<0.05 were enrolled in multivariate analysis. A nomogram model was constructed based on the variables considered statistically significant in multivariate analysis. The receiver operating characteristic (ROC) analysis, C-index value, and calibration plots were applied to measure the discrimination performance of the nomogram in both the training cohort and validation cohort.

All the statistical analyses were performed using the Stata version 15.1(StataCorp, College Station, TX) and R version 3.5.1. Differences were considered statistically significant when the two-tailed *P* value was less than 0.05.

## 3. Results

### 3.1 Clinicopathological characteristics

Our study consisted of 110 patients (95 men and 15 women), including 103 primary liver cancer patients and 7 secondary liver cancer patients. The median age was 58 (51-66) years old. Most of the patients (98/110) underwent chemoembolization through the left radial artery. Twelve patients underwent chemoembolization through the right radial artery. Patients were randomly divided into a training set (n=55) and a validation set (n=55). The mean total fluoroscopy time and time to angiography fluoroscopy were 5.34±4.07 min and 2.81±3.20 min, respectively. The patients' clinicopathological characteristics were shown in Table 1.

### 3.2 Risk factors

To explore risk factors for TRA chemoembolization in patients with liver cancer, univariate and multivariate analyses were performed. The cut-off value of total fluoroscopy time was 6 min determined by the upper tertile. In the training cohort, the univariate analysis indicated that catheter type, vascular variation, Myla classification, the length from LSA to TA, the distance from LSA to TA, and the angle between CHA and abdominal aorta were associated with total fluoroscopy time of transradial approach chemoembolization. Multivariate analysis demonstrated that vascular variation (OR=3.01, 95% CI: 1.13-8.07, *P*=0.028), Myla classification (OR=2.51, 95% CI: 1.09-5.75, *P*=0.030), the distance from LSA to TA (OR=5.71, 95% CI: 1.37-23.85, *P*=0.017) and the angle between CHA and abdominal aorta (OR=0.17, 95% CI: 0.04-0.73, *P*=0.017) were independent factors predicting technical complexity of TRA chemoembolization (Table 2), indicated by total fluoroscopy time over 6 min.

### 3.3 Development and validation of CTA-based nomogram

**Figure 2:**
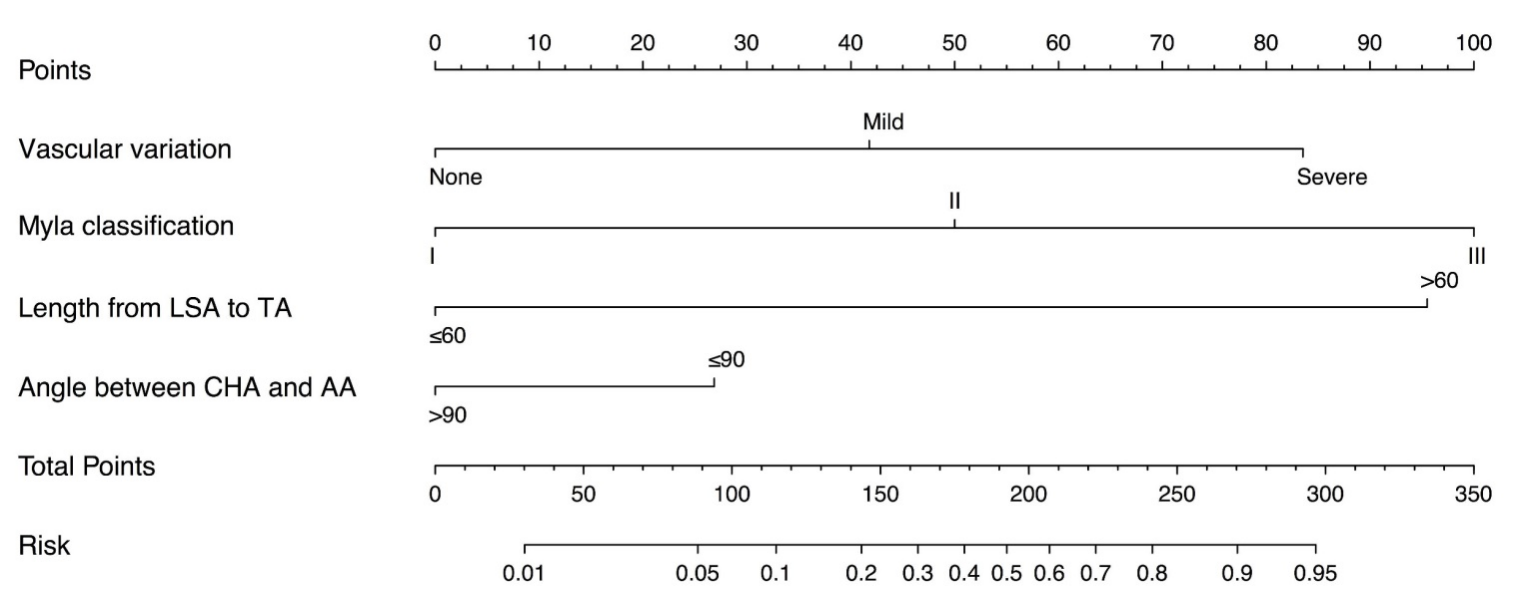
Nomogram predicting total fluoroscopy time of transradial approach chemoembolization for patients with liver cancers.

**Figure 3:**
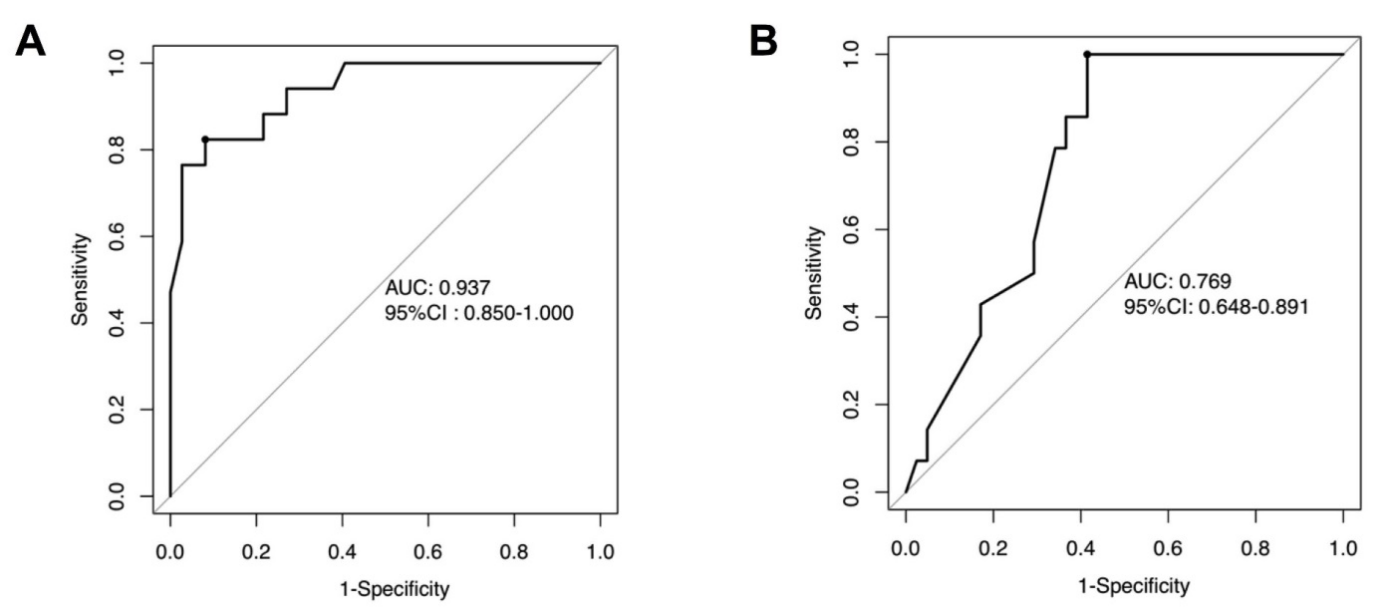
Receiver operating characteristic (ROC) curves of the nomogram in the training cohorts (A) and the validation cohort (B).

**Figure 4:**
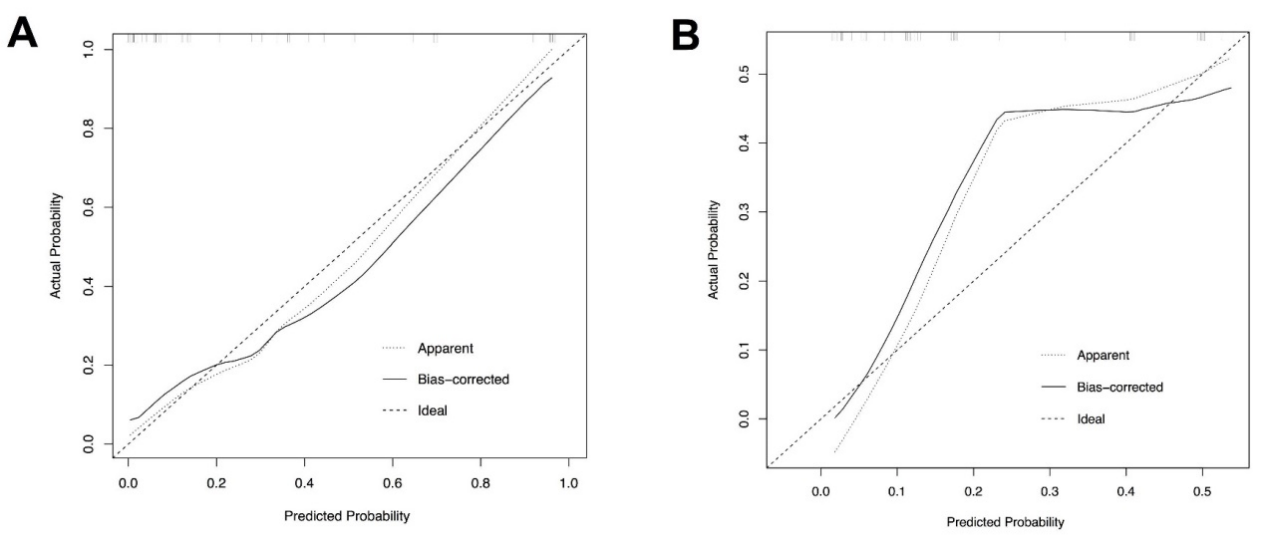
Calibration plots of the nomogram to predict total fluoroscopy time in the training cohort (A) and the validation cohort (B).

Based on the independent risk factors identified in multivariate analysis in the training cohort, we constructed a CTA-based nomogram model to predict the technical complexity of TRA chemoembolization for individual patient. To facilitate clinical usage, each variable was assigned a score according to its β coefficients (Figure 2) in our model. The newly developed nomogram model had a high C-index value of 0.928 in the training cohort and 0.827 in the validation cohort (Table 3). The AUC values also demonstrated satisfactory prediction ability of the present nomogram (Training cohort: AUC=0.929; Validation cohort: AUC=0.769) (Table 3, Figure 3). Moreover, the calibration curves for predicting total fluoroscopy time indicated that our nomogram was well-calibrated in both training cohort and validation cohort (Hosmer-Lemeshow test *P*=0.618 and 0.299, Figure 4).

### 3.4 Optimal cut-off value of the nomogram

The optimal threshold value of the nomogram was determined as 150 points based on the ROC analysis in the training cohort. Patients were divided into low-complexity group (score ≤150 points) and high-complexity group (score >150 points). The performance of the nomogram stratification was illustrated in Table 3. In the entire cohort, the rates of total fluoroscopy time > 6 min were 12.5% in the low-complexity group and 57.9% in the high-complexity group, respectively. In the training cohort, the rates of total fluoroscopy time > 6 min were 8.1% in low-complexity group and 77.8% in high-complexity group, respectively (Table 3). In the training cohort, the total fluoroscopy time was 9.22±6.20 min in the high-complexity group and 3.71±1.45 min in the low-complexity group (*P*<0.001), respectively. In the validation cohort, the total fluoroscopy time was 7.02±4.95 min in the high-risk group and 4.12±1.99 min in the low-complexity group (*P*=0.003), respectively.

## 4. Discussion

Evidence from previous studies 2, 3, 17 has demonstrated the technical feasibility and safety of TRA chemoembolization in patients with liver cancer. Based on our initial experience, including patient selection, technical nuances, and the learning curve of TRA chemoembolization, we found that some patients were unsuitable for TRA chemoembolization due to their atypical vascular morphology. Therefore, evaluation and selection of appropriate candidates are essential for the wide use of TRA chemoembolization in patients with liver cancer. In the present study, we showed that radiographic signatures of thoracoabdominal aorta CTA were capable to predict the technical complexity of TRA chemoembolization. Then, we developed and validated a CTA-based nomogram model, which displayed high accuracy in predicting the fluoroscopy time of TRA chemoembolization. This novel nomogram can serve as a non-invasive approach to identify ideal candidates for TRA chemoembolization in clinical practice.

During the construction of the nomogram, we found that four features were independently associated with long fluoroscopy time during TRA chemoembolization: vascular variation (*P*=0.028), Myla classification (*P*=0.030), length from LSA to TA (mm) (*P*=0.017), and angle between CHA and abdominal aorta (*P*=0.017). Our multivariate analysis showed that the presence of vascular variation significantly increased the difficulties of TRA procedure, indicated by prolonged fluoroscopy time. Due to the long route from the radial artery to hepatic artery and sharper bends in the route, the vascular variation, such as vascular distortion, vascular slenderness, and differences in vascular structure made TRA chemoembolization more difficult to perform compared with TFA chemoembolization. Myla et al divided the aortic arch morphology into three types, and this classification has been widely used to guide cardiovascular surgery and interventional procedure 15. Our study showed that type III aortic arch usually presented with more technical difficulties during TRA procedure and took longer fluoroscopy time compared with other types of aortic arch. This finding was consistent with what had been reported in carotid artery stenting procedures 18. Moreover, we found that the length from LSA to TA was also related to fluoroscopy time, due to small aortic arch angles as well as distortion of the aortic arch. In addition to the abovementioned factors, the angle between CHA and the abdominal aorta also had great relevance with fluoroscopy time. The small angle between CHA and abdominal aorta significantly increased the complexity for the operators to insert the catheter into the hepatic artery and subsequent super-selection of tumor-supplying vessels.

Procedural techniques for engaging the common hepatic arteries and performing interventions vary significantly according to the operator's preferences and experiences. In our study, a vascular introducer sheath (5-Fr) and a single-catheter (regularly 4-Fr) technique was used in the operation. Regarding the selection of catheters, both 5-Fr and 4-Fr guide catheters can be chosen when a 5-Fr vascular sheath is inserted into the radial artery. In our center, 4-Fr catheters are preferable in TRA procedure because they are more flexible and maneuverable compared with 5-Fr catheters, especially for patients with peripheral arterial stenosis or distortion indicated by thoracoabdominal aorta CTA. Our initial experience showed that the overall operation success rate is generally higher with 4-Fr guide catheters than that with 5-Fr catheters. Although 4-Fr catheters are preferable in TRA chemoembolization, a 5-Fr sheath is still recommended in TRA procedure due to its low incidence of radial artery occlusions and convenience for operators to change catheters. Actually, previous studies have revealed that 5-Fr sheaths had a low incidence of radial artery occlusions (RAO) (1.1%) 19, and a low incidence of severe reduction in radial artery flow 20-22.

In the past, since patients' vascular conditions could not be assessed before operations, the operators could only select catheters according to the angiography during the procedure, which may lead to catheter exchange. We constructed this CTA-based nomogram model to help the operators to evaluate patients' vascular conditions before operations. Based on our model, interventionalists could utilize different techniques to reduce exposure time and increase success rates of TRA chemoembolization. As we know, during TRA chemoembolization procedures, there are two important angles en route from the radial artery to the common hepatic artery, that is, the angle between LSA and aortic arch and the angle between the abdominal aorta and common hepatic artery. Thus, the technical complexity of TRA chemoembolization lies in two aspects: turning over the aortic arch and hepatic artery super-selection. Myla classification, the angle between LSA and aortic arch, length from LSA to TA, and distance from LSA to TA could reflect the degree of aortic arch distortion, whereas the angle between CHA and the abdominal aorta is associated with the degree of hepatic artery distortion. These variables were included in the nomogram in our study. Based on our nomogram, patients were divided into low-complexity group (score ≤ 150 points) and high-complexity group (score >150 points). Patients in high-complexity group may not be ideal candidates for TRA chemoembolization because of high technical complexity. For patients classified in high-complexity group, we propose to apply a long microcatheter (150 cm) during hepatic artery super-selection. A long microcatheter can increase the tension of the guiding catheter and maintain the stability of the guiding catheter, thus improving success rates of super-selection. For these patients who are usually with a sharp angle between LSA and TA or a sharp angle between CHA and abdominal aorta (<90), a Cobra catheter or a TIG catheter is recommended due to a bigger angle of the catheter tip in comparison with the MPA catheter.

This clinical model achieved satisfactory predictive efficacy indicated by AUC and C-index. Moreover, good calibration was confirmed by the Hosmer-Lemeshow test illustrated in Figure 4. To our knowledge, this nomogram is the first clinical model contributing to identifying ideal candidates for TRA chemoembolization in patients with liver cancer.

The present study has some limitations. Firstly, as a retrospective study, patient selection bias was inevitable. Moreover, TRA chemoembolizations in this study were conducted by different interventionalists, which inevitably brought about a disparity in total fluoroscopy time. In addition, the nomogram model was established based on the Asian patient cohort and might not be directly extrapolated to Western patients before external validation. Future large sample size, multicenter, prospective study is warranted.

## 5. Conclusion

We develop and validate a CTA-based nomogram model to individually predict technical complexity of TRA chemoembolization in patients with liver cancer. The newly established model may help to identify ideal candidates for TRA chemoembolization and guide clinical decision-making.

## Figures and Tables

**Table 1:** Characteristics of the patients in the training and validation cohorts

Variable	No. of patients (%) or Median (IQR)		
	Entire cohort (N=110)	Training cohort (N=55)	Validation cohort (N=55)
Age	58(51-66)	56(46-65)	62(55-66)
Sex			
-Male	95(86.4)	47(85.5)	48(87.3)
-Female	15(13.6)	8(14.5)	7(12.7)
Height	170(165-173)	170(163-172)	170(162-174)
Weight	64(55-70)	67(55-71)	60(56-65)
AFP	46.4(6.2-1815)	111.5(5.5-14563)	29.6(6.3-312.7)
Chronic disease			
-No	86(78.2)	46(83.6)	40(72.7)
-Yes	24(21.8)	9(16.4)	15(27.3)
Tumor type			
-Primary HCC	99(90.0)	46(83.6)	53(96.4)
-Primary ICC	4(3.6)	4(7.3)	0(0.0)
-Secondary liver cancer	7(6.4)	5(9.1)	2(3.6)
HbsAg			
-Negative	22(20.0)	13(23.6)	9(16.4)
-Positive	88(80.0)	42(76.4)	46(83.6)
Catheter type			
-MPA	96(85.5)	46(83.6)	48(87.3)
-Other	16(14.5)	9(16.4)	7(12.7)
Vascular variation			
-None	83(76.2)	37(68.5)	46(83.6)
-Mild	6(5.5)	4(7.4)	2(3.6)
-Severe	20(18.4)	13(24.1)	7(12.7)
Myla classification			
-I	39(35.5)	22(40.0)	17(30.9)
-II	29(26.4)	11(20.0)	18(32.7)
-III	42(38.2)	22(40.0)	20(36.4)
Angle between LSA and aortic arch	73(62-82)	72(63-80)	75(60-83)
Length from LSA to TA (mm)	61(51-72)	60(51-71)	63(51-74)
Distance from LSA to TA (px)	71(46-96)	65(34-92)	79(56-99)
Angle between CHA and abdominal aorta	85(65-115)	83(65-110)	87(67-118)
Total fluoroscopy time (min)	4.3(3.2-6.1)	4.3(3.2-6.2)	4.3(3-6.1)

Abbreviations: IQR: interquartile range; N: number; AFP: alpha fetal protein; HCC: hepatocellular carcinoma; ICC: intrahepatic cholangiocarcinoma; LSA: left subclavian artery; TA: thoracic aorta; CHA: common hepatic artery.

**Table 2:** Univariate and multivariate analysis of total fluoroscopy time in the training cohort

	Univariate analysis		Multivariate analysis	
	OR (95% CI)	*P* value	OR (95% CI)	*P* value
Age (>65)	0.84(0.25-2.80)	0.781		
Sex (male)	0.68(0.16-2.80)	0.590		
Height (>170)	1.31(0.47-3.68)	0.606		
Weight (>60)	0.89(0.31-2.54)	0.823		
AFP (>400)	1.91(0.69-5.35)	0.215		
Chronic disease (yes)	1.23(0.35-4.37)	0.751		
Tumor type	0.41(0.17-1.03)	0.058		
HbsAg (positive)	1.57(0.46-5.35)	0.475		
Catheter type	33.47(3.71-301.86)	**0.002**		
Vascular variation	4.33(2.04-9.22)	**<0.001**	3.01(1.13-8.07)	**0.028**
Myla classification	4.38(2.07-9.25)	**<0.001**	2.51(1.09-5.75)	**0.030**
Angle between LSA and aortic arch	2.91(0.83-10.23)	0.096		
Length from LSA to TA (>60 mm)	11.14(3.08-40.31)	**<0.001**	5.71(1.37-23.85)	**0.017**
Distance from LSA to TA (>90 px)	13.17(3.25-53.33)	**<0.001**		
Angle between CHA and abdominal aorta (>90)	0.12(0.03-0.42)	**0.001**	0.17(0.04-0.73)	**0.017**

Abbreviations: OR: odds ratio; CI: confidence interval; AFP: alpha fetal protein; LSA: left subclavian artery; TA: thoracic aorta; CHA: common hepatic artery.

**Table 3:** Performance of the nomogram in predicting total fluoroscopy time in the training and validation cohorts

Performance parameter	Training cohort	Validation cohort
AUC	0.929	0.769
95% CI low	0.850	0.648
95% CI high	1.000	0.891
C-index	0.928	0.827
Specificity	0.8947	0.7073
Sensitivity	0.8235	0.5714
Positive-LR	10.1569	2.3010
Negative-LR	0.1920	0.3254
Positive-PV	0.78	0.40
Negative-PV	0.92	0.83

Abbreviations: AUC: Area Under the Curve; CI: confidence interval; LR: likelihood ratio; PV: predictive value.

## Abbreviations

CTA: angiography computed tomography
TAE: transcatheter arterial embolization
TACE: transcatheter arterial chemoembolization
TFA: transfemoral approach
TRA: transradial approach
HCC: hepatocellular carcinoma
ICC: intrahepatic cholangiocarcinoma
PS: performance status
AFP: alpha fetal protein
LSA: left subclavian artery
TA: thoracic aorta
CHA: common hepatic artery
OR: odds ratio
IQR: interquartile range
ROC: receiver operating characteristic
AUC: area under the curve
CI: confidence interval
LR: likelihood ratio
PV: predictive value
